# Evidence of differential phenotypic plasticity in a desert mustard

**DOI:** 10.1002/ece3.10479

**Published:** 2023-08-31

**Authors:** Brian Alfaro, Diane L. Marshall

**Affiliations:** ^1^ Department of Biology Eastern University St. Davids Pennsylvania USA; ^2^ Department of Biology University of New Mexico Albuquerque New Mexico USA

**Keywords:** adaptation, climate change, crop‐wild relatives, epigenetics, phenomics

## Abstract

Understanding the effect of the environment on trait variation is critical for ecologically and economically important plants. Here, we asked whether differences in soil moisture are a source of variation in Sahara mustard (*Brassica tournefortii*). We subjected common garden populations of plants derived from native, invasive, and landrace sources (ranges) to varying water addition treatments. Using principal component analysis, we generated composite variables of life history traits for ANCOVA tests and plotted norms of reaction. Planting time was included as a covariate because we observed differences in seedling emergence despite efforts to standardize germination. We also examined the population coefficient of variation of individual traits (plasticity) and the association of trait CVs with fitness. The amount of plasticity varied but was inconsistent among range sources for all composite traits. Planting time did not affect treatments, but plants from different ranges responded differently to variable planting times. With a surplus of water, plants derived from native and invasive populations plateaued in vegetative trait values but showed a continuous linear increase in reproductive trait values. Possibly as a result of domestication, moderate and high water treatments in landrace plants caused plateaus in composite trait values for flowering phenology, seed count, plant size, and branching. The ecological breadth shown by our plants is likely due to drought tolerance that evolved in *Brassica tournefortii* source populations.

## Graphical abstract

Our study investigates whether experimental lines derived from native, invasive, and landrace *B. tournefortii* populations exhibit divergent phenotypic responses under different soil moisture conditions. Some traits increase in value, and some traits plateau, depending on the type of character measured and where the plants were sourced originally.

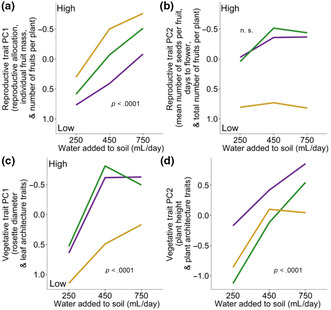

## 1 INTRODUCTION

Water affects a plant's life history. That is, a plant's traits interact with moisture in the environment for initial establishment, growth, metabolism, and reproduction (Nicotra & Davidson, 2010). These potential interactions between life history traits and environment can be categorized as phenotypic plasticity when a genotype produces multiple phenotypes from different environmental conditions (Bradshaw, 1965; Schlichting & Wund, 2014). By measuring traits expressed across an array of conditions, such as soil moisture, plasticity can be experimentally measured (Schlichting, 1986). The experimental phenotypic signatures of plasticity are illustrated via reaction norms, which are important in assessing environmental sources of variation in organisms (Richards et al., 2006).

For species that are sensitive to moisture, such as desert plants and crops, a positive phenotypic response to water in the soil can be a factor for successful colonization or increased yield (Nicotra & Davidson, 2010). The potential ecological breadth associated with plasticity to critical environmental factors, such as soil moisture, can be an advantage to recently introduced or domesticated populations with low genetic variation that exhibit plasticity. Conversely, well‐established wild or crop populations can have locally adapted traits that may not be highly plastic (Baker & Stebbins, 1965; Kusmec et al., 2018). Plastic response can therefore be associated with fitness, which makes trait plasticity itself a potential target for selection in wild or domesticated populations (Scheiner, 1993; Schlichting, 1986; Schlichting & Wund, 2014; Sultan, 2000). Breeders select for consistent yields from a diverse gene pool, potentially including plasticity that results in several genotypes with similar phenotypes (Connor et al., 2011). High plasticity, which leads to variable yield traits, can translate into increased overall yield because of the sensitivity of highly plastic genotypes to field treatments such as soil amendment and irrigation (de Felipe & Prado, 2021; Peltonen‐Sainio et al., 2011; Sadras et al., 2009). While breeders homogenize gene pools of commercial crops continually, landraces can have adaptive variability due to gene flow or seed sharing among growers (Mercer & Perales, 2010; Meyer & Purugganan, 2013). For invasive plants, two common scenarios for phenotypic plasticity are possible. First, trait plasticity can evolve as a neutral or nonadaptive trait that can result in phenotypic variation not associated with improved fitness (Lande, 2015; Matesanz et al., 2010). Second, phenotypic plasticity may provide sufficient adaptive variability so that the plants can overcome novel environmental conditions (Nicotra et al., 2010).

Despite the potential adaptive advantages of positive phenotypic responses to increasing moisture and high plasticity, high variability in moisture does not always associate with high plasticity in plants. There are possible causes for these mixed results (reviewed in Nicotra & Davidson, 2010). Variable plasticity among experimental groups can be caused by differences in the original local conditions of experimental plant sources that can result in local adaptation in the plastic response to moisture. Another related reason is that natural or artificial changes in the gene pool can cause differing patterns of plasticity among populations or among plant ranges.

Here, we quantify the plastic response to soil moisture for a species that, in addition to wild populations, is a crop and an invasive plant. Specifically, we experimentally identified the types of traits that show variability due to soil moisture, and measured plasticity and its effect on the performance of populations bred from different sources, including native, invasive, and agricultural sources. We examine *Brassica tournefortii*, a xerophytic mustard native to North Africa, Europe, and the Middle East, that is cultivated as a seed crop in India and Pakistan (Rao et al., 1996) but is considered invasive in western North America and Australia (Bangle et al., 2008; Barrows et al., 2009; Boutsalis et al., 1999; Schiermeier, 2005; Trader et al., 2006). In the 1920s, this species was accidentally introduced from the soil surrounding date palms from the Middle East transported to Mecca, California (Dimmitt, 2009). The plant was deemed invasive in Northern America in the last two decades due to its impact on both state and federal wild lands and on human infrastructure (Barrows et al., 2009).

*Brassica tournefortii* plants sourced from different ranges differed in adaptive potential, and their population trait means varied with aridity in *B. tournefortii* among sources (Alfaro & Marshall, 2019). Because aridity was a factor in plasticity in these plants (see Nicotra & Davidson, 2010), we further investigated the phenotypic response to soil moisture treatment in different field collections and accessions of *B. tournefortii*. Analyzing the plastic response to soil moisture in *B. tournefortii* can test whether water can be a critical limiting factor in a desert annual species (Potter, 2019).

Specifically, we asked whether *B. tournefortii* grown in a common garden, produced from seeds developed in a greenhouse, and sourced from native, invasive, and landrace ranges will differentiate in trait means, have different patterns of reaction norms, and have different amounts of plasticity in response to variation in soil moisture. If so, then trait plasticity via soil moisture is a factor for phenotypic differentiation in these experimental populations. Also, we evaluated trends in the amount of plasticity and relative fitness for individual traits and tested for differences in these associations among experimental populations of *B. tournefortii* sourced from native, invasive, and landrace ranges. If plants from a specific source range showed a significant association between the amount of plasticity and fitness, then we considered the association to have adaptive significance. We designed our study using four hypotheses:

Hypothesis 1 Mean trait values will vary among experimental populations sourced from native, invasive, and landrace source ranges (hereafter, ranges). If the mean trait values of native, invasive, and landrace *B. tournefortii* are measured in a common environment (Alfaro & Marshall, 2019; Winkler et al., 2018), we predict the three ranges will exhibit different mean trait values in the face of varying levels of abiotic stress from soil moisture treatments.

Hypothesis 2 Reaction norms for plasticity will differ among the experimental populations of native, invasive, and landrace ranges of *B. tournefortii*. Invasive *B. tournefortii* in the wild grows and spreads rapidly in years when mean annual precipitation is higher than average (Li et al., 2015). We therefore expected the experimental populations of invasive and native plants to respond similarly to increasing water treatments. *B. tournefortii* does not increase its range during drought years in the southwestern United States (Li et al., 2015). Therefore, we predicted that experimental populations sourced from native and invasive ranges would increase in phenotypic values with increases in water but respond negatively to water deficits. We expected that, due to artificial selection for consistent yield, experimental populations of landrace *B. tournefortii* would express larger trait values, especially for yield traits, when water availability was increased and remain constant in trait values when water was scarce.

Hypothesis 3 The amount of plasticity of individual traits measured as population coefficients of variation will differ among the native, invasive, and landrace ranges. Using a subset of individuals from each range, we found that, for two polymorphic microsatellites, experimental plants from the invasive range had lower heterozygosity than native and landrace ranges (Alfaro, 2021). Because of the moderate to high phenotypic variation (Alfaro & Marshall, 2019) and low genetic diversity in experimental plants from the invasive range, we predicted that plants from the invasive range would have higher amounts of plasticity than plants from the native and crop ranges.

Hypothesis 4 Plasticity of individual traits will be associated with fitness in experimental populations, but trends will differ among native, invasive, and landrace ranges. For the native‐sourced *B. tournefortii*, phenotypic variability is more likely due to local adaptation that occurred in the source environment than to plasticity because it has existed in its habitats for millennia. We therefore expected a negative trend for fitness versus plasticity in the native range. Landraces are selected for consistent harvest and larger yields, so we expected a flat trend of plasticity versus fitness in the experimental populations. Lower genetic diversity in the invasive range (Alfaro, 2021; Winkler et al., 2018) will result in plasticity having a larger effect on the increase of phenotypic variation. Therefore, we expected fitness to increase with increasing plasticity in the invasive plants.

## 2 METHODS

### 2.1 Experimental populations

**FIGURE 1:**
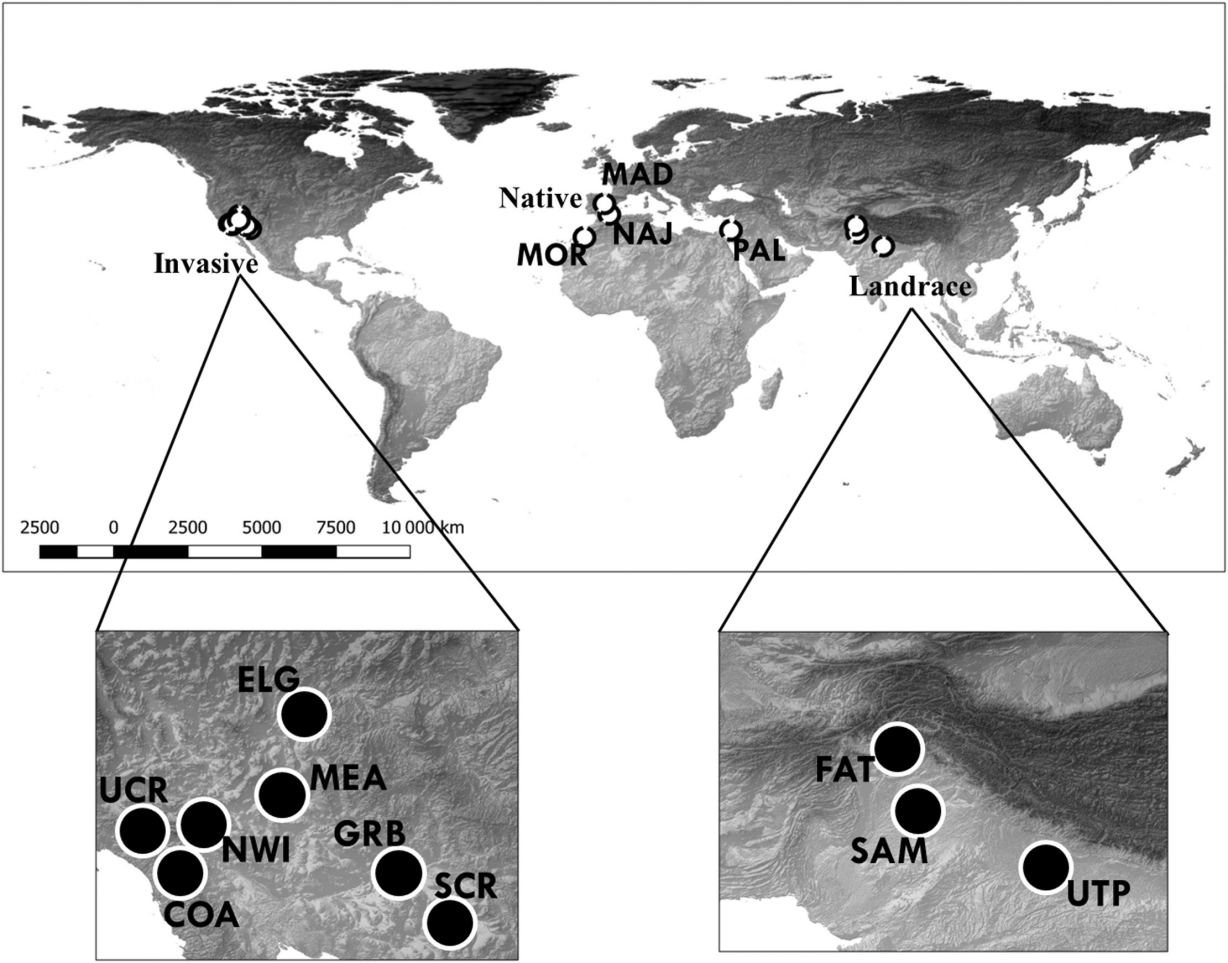
Map of original field collection sites for native and landrace seed accessions and invasive localities. The seed accessions from the native localities were collected from Tiznit (Morocco), Madrid (Spain), Almeria (Spain), and Palmachim (Israel). The seed accessions from the agricultural localities were from Sammundri (Pakistan), Fateh Jang (Pakistan), and Uttar Pradesh (India). Seeds from the invasive localities were field collected in the southwestern United States from Coachella Valley (CA), North Indian Canyon Rd., (CA), near the University of California, Riverside (CA), Santa Cruz River (AZ), Gila River Basin (AZ), near Elgin Road (NV), and Lake Mead (NV).

We developed experimental populations using seeds derived from native, invasive, and landrace populations. We propagated six experimental populations via artificial crosses from seeds sampled from invasive localities of *B. tournefortii* (Figure 1) in the Chihuahua, Sonora, and Mojave deserts in the southwestern United States. This invasive range originated from one introduction in the 1920s (Winkler et al., 2018), so axiomatically, these localities have undergone an initial sampling effect that was followed by decades of selection in the wild. Seeds from invasive populations were collected in 2008, and we began developing the experimental populations in 2014. We requested two groups of seeds from the U.S. Department of Agriculture—Agricultural Research Services (USDA‐ARS) National Genetic Resources Program accessions; one group from wild or native locations and another from cultivated plants identified as landraces. For each accession, USDA biologists randomly collected seeds from multiple plants at native, invasive, and landrace collection sites. These field collections were grown and maintained to produce seeds from sampled localities that can be distributed to researchers. We propagated seven experimental populations from genetic lines that we produced from native and landrace accessions. Seeds from all three ranges spent the same amount of time in storage. Based on their histories, both field and accession sources may have experienced some form of genetic bottleneck and unintentional selection before we produced our own experimental populations. Since the native and landrace accessions were maintained by the same protocols, they may have become more similar over the time they were propagated and stored by the USDA. However, our further procedures, see below, ensured that the seeds we used for our experimental populations were all bred in the same greenhouse at the same time. Thus, the immediate environmental effects on seed development and seed age were the same for all experimental populations.

**FIGURE 2:**
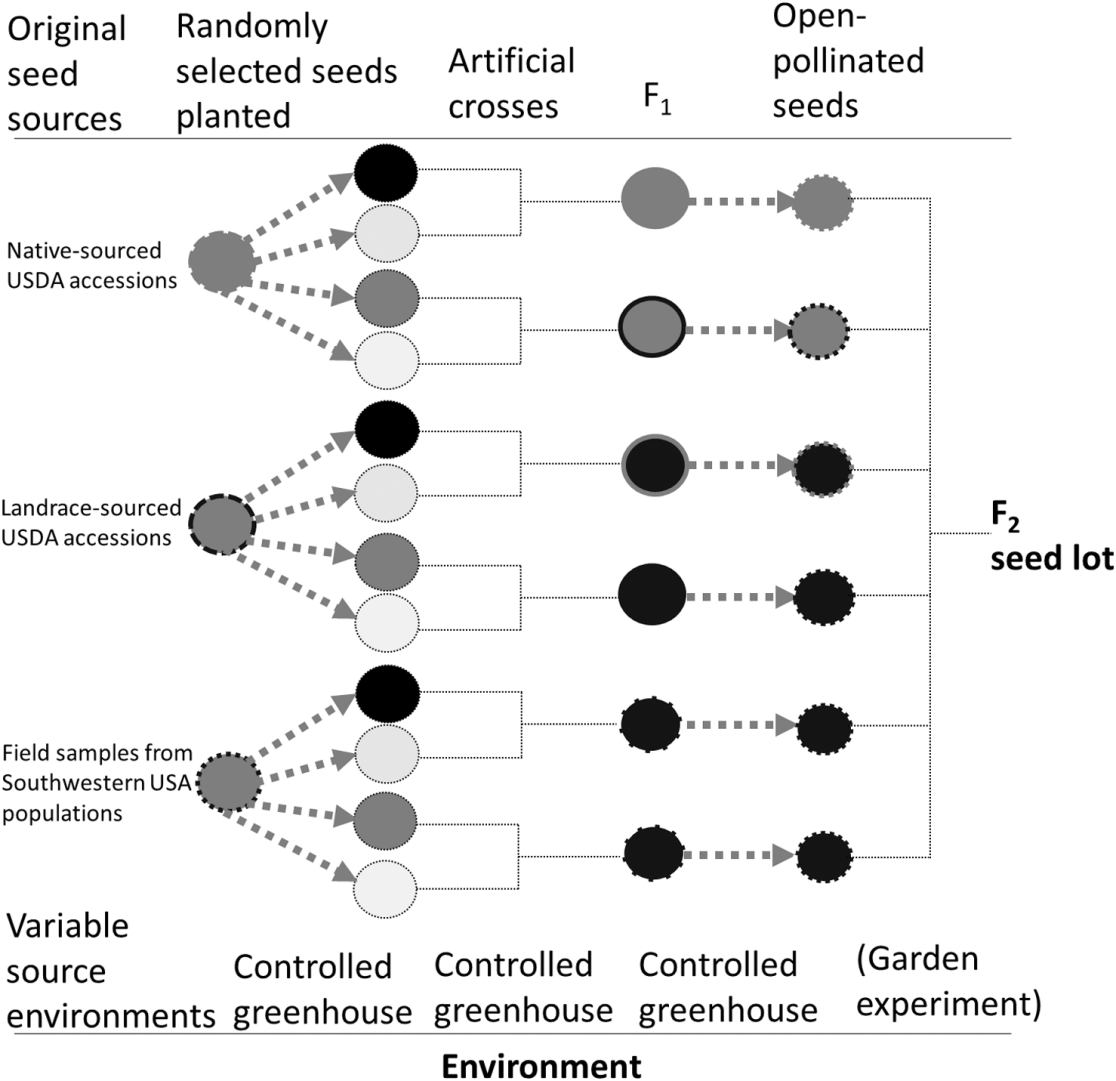
Plant husbandry pedigree of seed families used in the experiment. Two families per range type are shown as examples. Invasive plants were started from seeds that were collected in the southwestern United States during the 2008 growing season. Seeds were requested from wild or native locations and from landrace agricultural populations; the collection dates for the native and landrace accessions are in Table S1.

In March 2015, we planted original field seed collections and USDA seed accessions to create a parental generation in a common environment. F_1_ seeds from artificial crosses (Figure 2) from the parental generation were grown in a common greenhouse (see Alfaro & Marshall, 2019 for details on artificial crosses). Bud and flower dissections confirmed that pollen shedding occurs before anthesis in *B. tournefortii*; cross‐fertilization was unlikely because the greenhouse had no insect pollinators. Despite the sampling effect and unintentional selection that collected seeds experienced, F_1_ generations bred from accessions and localities of *B. tournefortii* had sufficient variability for analyzing phenotypic differentiation (Alfaro & Marshall, 2019). Specifically, trait variation from experimental F_1_ generations showed clines of trait means and selection gradients that increased or decreased when regressed with aridity indices from source environments of native, invasive, and landrace *B. tournefortii* (Alfaro & Marshall, 2019). These results suggest that the clines formed by experimental F_1_ generations were from genotypes that evolved in their source environments. We allowed the F_1_ plants in Alfaro and Marshall's (2019) common greenhouse study to self‐fertilize to produce F_2_ seeds, which we used as seed families for this investigation.

We produced populations from seeds that were sourced from native, invasive, and landrace ranges so we could examine and compare experimental phenotypic responses. We understand that the accessions used to generate experimental native and landrace populations were sampled in different decades (Table S1); these samples could potentially have undergone unintended selection at the USDA facilities. Therefore, our findings represent and describe how our own lines of *B. tournefortii* responded to the soil moisture treatments we administered in a common garden.

### 2.2 Experimental design

To germinate the F_2_ seeds, we used plastic 100 × 15 mm Petri dishes lined with Whatman™ filter paper that were soaked with 2.5 mL of a 5% gibberellic acid solution (to ensure germination) before adding seeds. After 24 h, we planted three seedlings that had developed both radicle and cotyledons in each of the 3.78 liter pots containing a 1:1 mix of sand and Metro Mix® (SunGro Horticulture®, Canada). We thinned each pot to one seedling as the first leaves emerged. Some seed families were not viable or had longer germination times, even with hormone treatment. When an F_2_ seed family did not germinate enough replicate seedlings, we planted F_2_ seeds from a different plant from the F_1_ family. Due to late‐germinating or inviable seeds, planting times varied from 1 to 45 days from the start of the experiment (April 10, 2017), which we based on the average of the last frost dates from the last 2 years before the experiment. To correct for planting time variation, we included the time of planting as a variable in our analyses.

To include all 14 localities from the native, invasive, and landrace ranges used in our previous study, we designed this experiment with 14 localities × 4 families/locality × 3 watering levels × 3 blocks, *n* = 504 (1 plant/family/treatment/block). Using PROC PLAN in SAS 9.4 (SAS Institute Inc., Cary, NC), we used a stratified random arrangement of the pots and their treatments so each of the three blocks had one replicate of each locality × family × watering level treatment. The garden experiment was in a fenced vacant lot in Albuquerque, New Mexico, with an available water source. The seedlings grew in standardized conditions until the first two leaves matured. Seedlings were hand‐watered with 200 mL of tap water per day (100 mL at 7:00 am and 100 mL at 5:00 pm) and fertilized with Peters® 20–20–20 General Purpose fertilizer (The Scotts Company, Marysville, OH) once a week until the end of the experiment. In addition to the standard irrigation, after the first 2 weeks, we administered three water treatments at 7:00 am daily by hand‐watering pots using plastic beakers. We chose these specific amounts because they were within the range of precipitation for the origins of the study:

low −250 mL
medium −450 mL
high −750 mL.

In mid‐July, the ambient daily temperature and insolation had resulted in some mortality. To prevent further mortality, we erected a 4‐meter‐high shade constructed from sheet metal to reduce the amount of sunlight. When some plants experienced wilting, we administered an extra 150 mL of tap water to all plants, regardless of treatment.

### 2.3 Trait measurements

#### 2.3.1 Phenology

We measured the days from seedling emergence to the appearance of the first flower as an index of phenology because it could be tracked accurately and consistently. Previous experiments showed that this variable represents overall phenology reliably (Alfaro & Marshall, 2019).

#### 2.3.2 Plant size

We counted the number of leaves at the time of the appearance of the first flower and measured rosette size at 30 days after the first flowering by measuring the length between the tips of the two largest diametrically opposite leaves to the nearest 0.1 cm using a meter stick. We measured plant height at 30 days after the first flowering to the nearest 0.1 cm using a meter stick. We weighed the aboveground biomass for each plant by excising the entire aboveground portion of the plant at the base under the rosette and then placing each segment in a weighed paper bag. The plants were dried in an oven for 1 week at 65°C then weighed to the nearest 0.1 g.

#### 2.3.3 Plant architecture

Anecdotal field reports point to tumbleweed dispersal, or anemochory, as the main seed dispersal mode of *B. tournefortii* in its invasive ranges. This dispersal mode is affected by the architecture of branches (Baker, 2007). For *B. tournefortii*, the angle, length, and density of multiple degrees of lateral branching from the main stem of the plant contribute to a rhomboid plant architecture that can roll and bounce. At the time of seed dehiscence, plants can easily be abscised from the ground by strong winds (Alfaro pers. observation). After abscission, the entire plant becomes a diaspore that rolls and bounces for several 100 m while breaking off siliques in the process. We analyzed variability in these traits (Alfaro & Marshall, 2019), but branch length and number of branches were the two features that showed genetic basis and adaptive potential. Therefore, we included branch length and number of branches as trait variables for this study. At 30 days from the appearance of the first flower, we measured branch length for each plant by selecting the first lateral branch closest to the apical branch and measuring its length from the base to the tip with a metric ruler. To determine the number of branches for each plant, we counted the total number of branch tips per plant.

#### 2.3.4 Leaf traits

Leaf size: At 30 days from the first flower, we sampled two basal leaves from each plant. We excised each leaf's petiole from the basal stem region. Because leaves emerging during bolting are reduced in size, we haphazardly sampled two basal leaves between leaf four and leaf ten. We measured leaf length to the nearest 0.1 cm from the base of the petiole to the tip of the leaf blade using a metric ruler. We measured leaf width using a metric ruler to the nearest 0.1 cm at the lengthwise midpoint of the leaf blade. For each plant, we calculated the mean leaf length and mean leaf width from the two sampled leaves and used these measures as trait values.

Leaf margin morphology—we included the number of lobes per leaf because of its association with water‐related leaf stress and leaf metabolism. We measured the mean number of lobes per leaf per plant as an index of leaf margin morphology. For each plant, we counted the number of lobes for each sampled leaf and averaged the counts.

#### 2.3.5 Reproductive traits

Total number of fruits per plant: To measure the fecundity of each plant, we counted the total number of viable fruits per plant at 30 days after the appearance of the first flower.

Mean seed number per fruit: As a second variable for fecundity, we included the mean seed number per fruit for each plant. We selected three mature fruits located at the basal regions of haphazardly selected inflorescence branches and counted the total number of seeds in each individual fruit.

Individual fruit mass: To measure individual fruit mass, we used the three dried fruits used for seed counts. We weighed each fruit to the nearest 0.0001 g using a Mettler‐Toledo AG135 digital balance (Columbus, OH). We took fruit masses for all three fruits per plant (including seeds) and used the mean as a trait value for each sample to have a value for the mean mass of individual fruits, which we used to calculate reproductive biomass and other derived traits.

#### 2.3.6 Derived traits

Vegetative biomass: Vegetative biomass provided another variable for size in our experiment and was calculated by subtracting reproductive biomass from total aboveground biomass.

Reproductive allocation: For this study, we defined reproductive allocation as the percentage of aboveground biomass that was fruit biomass. We calculated this as total fruit biomass/aboveground biomass × 100. Reproductive allocation was arcsine square root transformed prior to analysis.

Relative fitness: We used relative fitness as an index of the performance of each individual plant relative to our entire study population. We calculated relative fitness by dividing each plant's total number of fruits by the maximum total number of fruits that we recorded in this experiment. We multiplied the values by 100 and then performed an arcsine square root transformation.

### 2.4 Statistical analysis

Before performing statistical tests, we asked whether each of the traits fit our assumptions for analysis of variance and regression analyses. Only two traits—relative fitness and reproductive allocation (calculated as a percentage of total biomass)—did not fit; these two traits were arc‐sin square root transformed. We performed box‐cox transformations in R Studio (*MASS* package) for all our trait variables. We did not include traits that were used to calculate derived variables, such as total aboveground biomass, to avoid multicollinearity.

To reduce the number of variables, we performed principal component analyses (PCA) for two trait groups, vegetative and reproductive, using the *prncomp* function in R on our data set. We ran these tests using built‐in functions in R Studio (R Core Team, 2018). We designated trait variables that have positive or negative loading values of at least 0.30 as highly loaded.

Using analysis of covariance (ANCOVA) and reaction norm approaches, we tested whether mean trait values varied among the ranges (Hypothesis 1) and whether phenotypic response increased or decreased with water supply among the three ranges (Hypothesis 2). All ranges had variation in planting time, so we included the time of planting relative to the average day of last frost (Planting Date) to detect whether planting time affected the plants' trait variation. We included Planting Date as a single term and as an interaction term with Soil Moisture Level and Range. We did not use automated variable or model selection but ran several models with different variable combinations and removed variables that were not significant across composite traits to conserve degrees of freedom. Specifically, we used the form: Trait Value = Days to Planting + Range + Volume of Water Added to Soil + Block + Population within Range + Block × Range + Range × Volume of Water Added to Soil + Days to Planting × Volume of Water Added to Soil + Days to Planting × Range. We used PROC GLM in SAS 9.4 (2013) for all ANCOVA models and Tukey HSD tests for pairwise comparisons among means (SAS Institute, Inc., Cary, NC). To compare the patterns of the effect of range and trait means among soil moisture treatments, we plotted reaction norms using the *ggplot2* package (Wickham, 2016) in R Studio (R Core Team, 2018). We visualized the effect of planting date as a covariate on the composite traits by invoking the generalized additive modeling method in *ggplot2*.

To test the association of plant plasticity with population fitness, we selected the two most highly loaded trait variables from each of our composite variables and calculated the coefficient of variation (CV = standard deviation/mean ×100) for the individual traits. After calculating population trait CVs, we performed type III ANOVA tests via PROC GLM in SAS 9.4 on each of the six listed traits and used range as a categorical variable to test Hypothesis 3. To compare the relationship of mean population plasticity CVs versus relative fitness among ranges (Hypotheses 4), we performed ANCOVA tests via PROC GLM in SAS 9.4 using the model: mean arcsine√ relative fitness = Range + Population Trait CV + Range × Population Trait CV. We plotted regression lines using the *ggplot2* package in R Studio (R Core Team, 2018).

## 3 RESULTS

### 3.1 Composite traits

After performing PCA, we narrowed our trait variables to four composite variables: Reproductive Trait PC1, Reproductive Trait PC2, Vegetative Trait PC1, and Vegetative Trait PC2. For Reproductive Trait PC1, the most highly loaded trait variables were reproductive allocation (−0.608), individual fruit mass (−0.576) and number of fruits per plant (−0.440). Reproductive Trait PC1 explained 36% of the variability in reproductive traits. Reproductive Trait PC2, which explained 25% of the variability in reproductive traits, was highly loaded for the mean number of seeds per fruit (−0.657), days to flower (−0.54), and number of fruits per plant (−0.424). Vegetative Trait PC1 explained 32% of variation in vegetative traits and was highly loaded for basal rosette diameter (−0.468), leaf length (−0.457), number of leaf lobes per leaf (−0.425), and number of leaves (−0.418). Vegetative Trait PC2 explained 26% of the variation in vegetative traits and was highly loaded for height (0.55), lateral branch length (0.542), and number of branches (0.422). The loadings for the PC axes' variables are listed in Table 1.

**TABLE 1:** Summary of composite trait variables via PCA.

Reproductive trait PC1	
Trait variable	Loading
Reproductive allocation	−0.61
Individual fruit mass per plant	−0.57
Total number of fruits per plant	−0.44
Mean seed number per fruit	−0.25
Days to flower	0.21
Reproductive trait PC2	
Trait variable	Loading
Mean seed number per fruit	−0.66
Days to flower	−0.54
Total number of fruits per plant	−0.42
Individual fruit mass per plant	0.11
Reproductive allocation	0.29
Vegetative trait PC1	
Trait variable	Loading
Rosette diameter	−0.47
Leaf length	−0.46
Number of leaf lobes	−0.43
Number of leaves	−0.42
Vegetative biomass	−0.33
Height	−0.22
Lateral branch length	−0.16
Total number of branches	−0.16
Vegetative trait PC2	
Trait variable	Loading
Height	0.55
Lateral branch length	0.54
Total number of branches	0.42
Vegetative biomass	0.21
Number of leaves	−0.06
Rosette diameter	−0.22
Number of leaf lobes	−0.22
Leaf length	−0.27

### 3.2 Trait variation among source ranges (Hypothesis 1)

**FIGURE 3:**
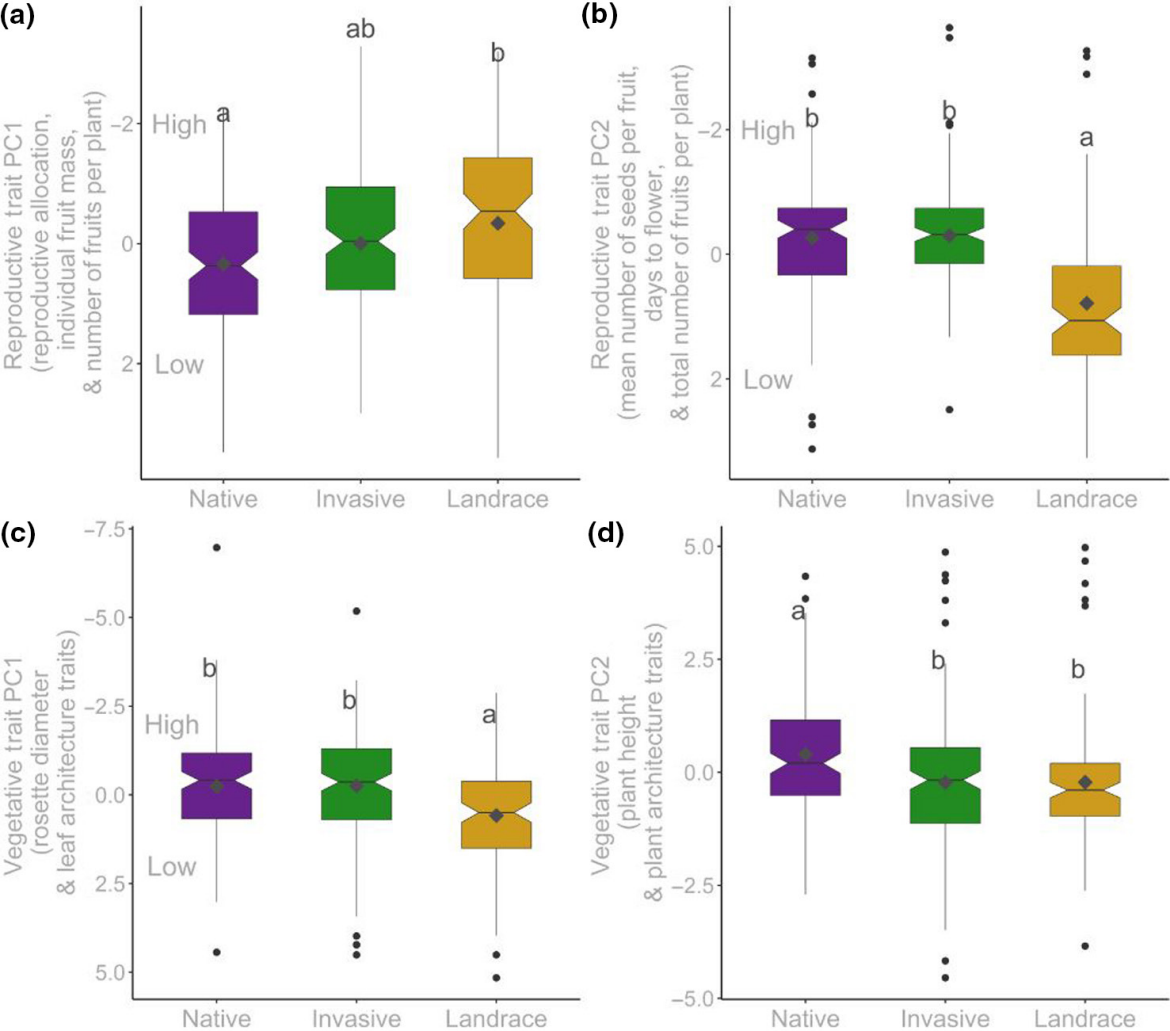
Box plots of trait principal components variables in native (*N* = 139), landrace (*N* = 115), and invasive (*N* = 168) ranges. Significantly different pairwise comparisons are indicated with Tukey HSD letter superscripts.

The experimental plants sourced from the landrace ranges showed the highest mean values for Reproductive Trait PC1, the composite trait highly loaded for reproductive allocation, individual fruit mass, and number of fruits per plant. The experimental invasive plants had the lowest mean trait value for Reproductive Trait PC1. These differences were statistically significant (Figure 3a, ANOVA results in Table 2). Reproductive Trait PC2, highly loaded for mean number of seeds per fruit, days to flower, and number of fruits per plant, significantly varied among ranges, with experimental landrace populations having a lower mean than other populations (Figure 3b, ANOVA results in Table 2). Mean Vegetative Trait PC1 (mostly leaf traits) did not differ between native and invasive source ranges; the landrace range, however, had a lower mean value than the invasive and native experimental populations (Figure 3c). These range‐wide differences were statistically significant (Table 2). Native plants had significantly larger values of Vegetative Trait PC2 (highly loaded for plant height, branch length, and number of branches) than invasive and landrace ranges (Figure 3d, Table 2).

**TABLE 2:** Summary of ANCOVA results that tested the effect of soil moisture levels on three principal components derived from 13 life history traits associated with reproduction, leaf morphology, and plant size and architecture (422 plants).

Source	Reproductive trait PC1 *R* ^2^ = .28			Reproductive trait PC2 *R* ^2^ = .30			Vegetative trait PC1 *R* ^2^ = .41			Vegetative trait PC2 *R* ^2^ = .38		
	*df*	*F*	*p*	*df*	*F*	*p*	*df*	*F*	*p*	*df*	*F*	*p*
Days to planting	1	8.20	**.004**	1	4.18	**.042**	1	104.33	**<.0001**	1	66.46	**<.0001**
Range	2	10.24	**<.0001**	2	12.88	**<.0001**	2	10.67	**<.0001**	2	10.56	**<.0001**
Volume of water added to soil	2	11.38	**<.0001**	2	0.48	.62	2	10.19	**<.0001**	2	15.43	**<.0001**
Block	2	5.55	**.004**	2	0.040	.96	2	16.48	**<.0001**	2	1.11	.331
Population within range	11	1.52	.12	11	1.93	**.035**	11	2.28	**.01**	11	1.98	**.030**
Block × range	4	2.16	.073	4	2.80	**.026**	4	0.51	.726	4	1.19	.315
Range × volume of water	4	0.43	.78	4	1.18	.32	4	1.29	.277	4	1.42	.228
Days to planting × volume of water	2	0.93	.39	2	0.62	.54	2	0.75	.472	2	2.54	.080
Days to planting × range	2	3.90	**.021**	2	3.16	**.043**	2	3.02	**.049**	2	4.96	**.008**

*Note*: Days to planting was used as a covariate to control for differences in planting time.

Bold values are the exact outputs from the statistical software.

### 3.3 Trait variation due to irrigation treatment (Hypothesis 2)

**FIGURE 4:**
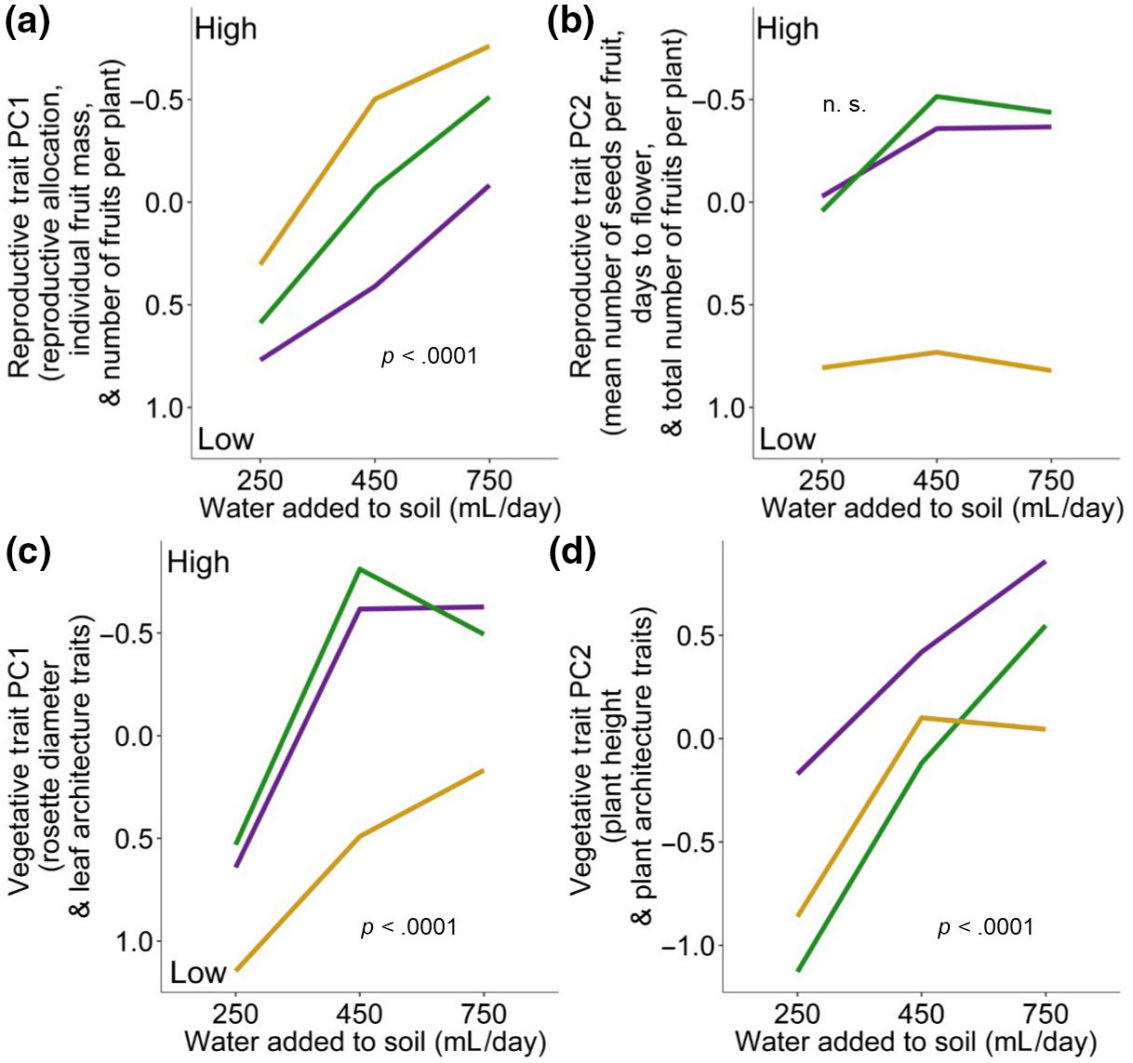
Reaction norms for four composite trait variables across three soil moisture treatments (volume of water added to soil) for experimental lines derived from native (*N* = 139), landrace (*N* = 115), and invasive (*N* = 168) populations. The *p* values of the effect of water treatments are shown (Purple = Native, Green = Invasive, Yellow = Landraces).

With increasing water, experimental plants from native and invasive ranges behaved similarly. They significantly increased in values for Reproductive Trait PC1 (reproductive allocation, fruit mass, and number of fruits per plant; Figure 4a, Table 2). Native and invasive plants tended to increase in trait values from low to moderate water additions and plateaued from moderate to high water additions for Reproductive Trait PC2 (mean seed number per fruit days to flower, and number of fruits per plant, Figure 4b), but there was no significant association between water treatment and trait values for this composite variable (Table 2). In native and invasive experimental plants, Vegetative Trait PC1 (rosette size and leaf traits, Figure 4c) plateaued in moderate to high water treatments, and Vegetative Trait PC2 (plant height and architecture traits) significantly increased linearly with increasing water additions in native and invasive populations (Figure 4d). The effects of water treatment on Vegetative Trait PC1 and Vegetative Trait PC2 are statistically significant (Table 2).

Although none of the range‐by‐treatment effects were statistically significant, landrace plants tended to show different patterns than the other ranges. Experimental landrace plants statistically increased in a linear fashion with increasing water for Reproductive Trait PC1 (reproductive allocation and fruit mass, Figure 4a, Table 2) and for Vegetative Trait PC1 (rosette size and leaf traits, Figure 4c, Table 2). Landrace plants showed a stable phenotypic response from low to high water additions for Reproductive Trait PC2 (mean seed number per fruit and days to flower), but there was no significant association between water treatment and trait values for this composite variable (Figure 4b, Table 2). Landrace plants increased in values of Vegetative Trait PC2 in a strong, linear fashion from low to moderate amounts of irrigation and did not change in phenotypic means from moderate to high amounts of water treatments (Figure 4d).

### 3.4 Other sources of variation and the effect of planting time

**FIGURE 5:**
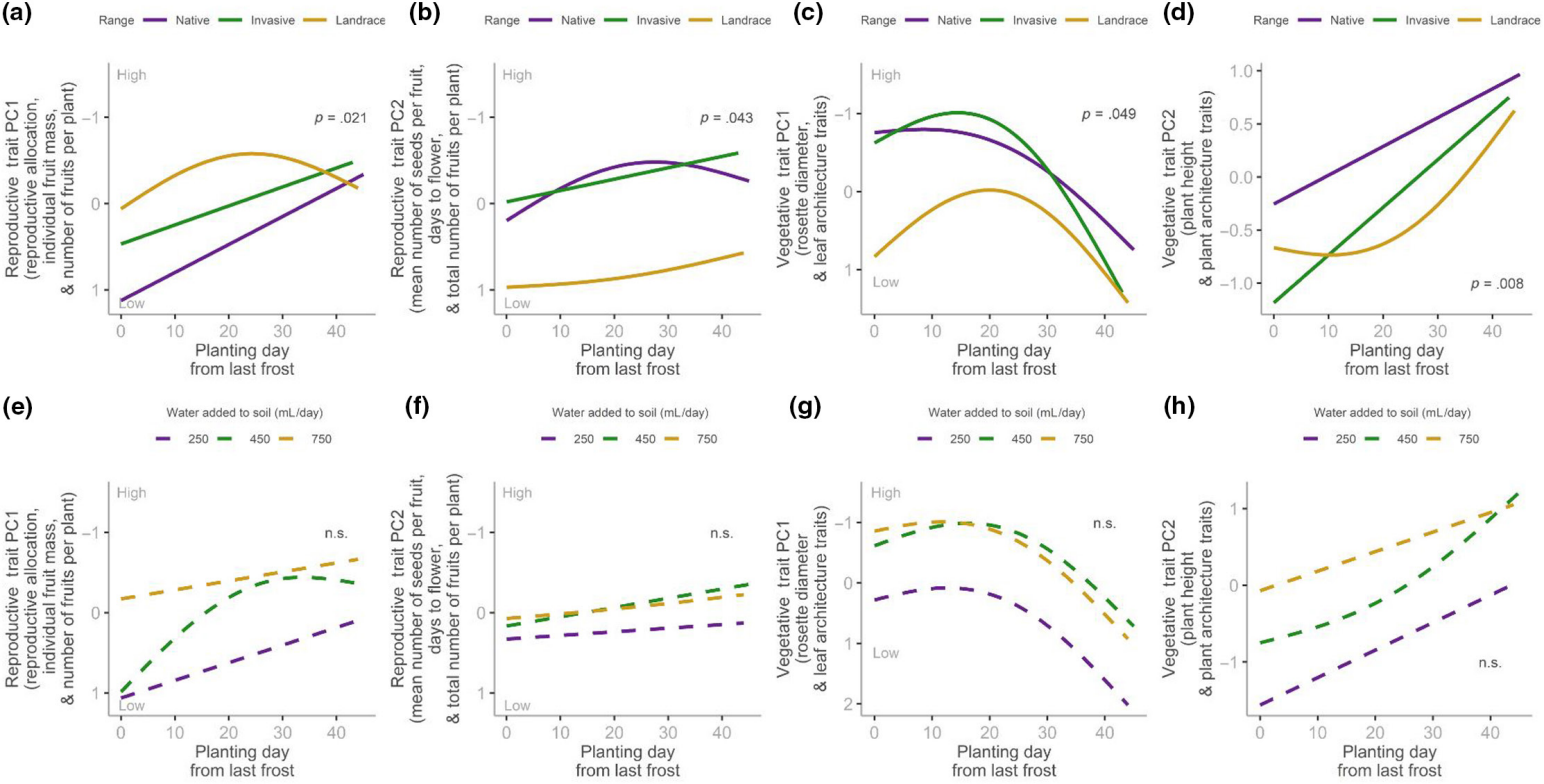
Regression lines smoothed via generalized additive models (*k* = 3) for four composite trait variables showing Days to Planting (planting days after frost) versus composite trait variables among source ranges (a–d) and Days to Planting versus trait variables among treatments (Volume of Water Added, e–h). ANCOVA P values for the interaction of Days to Planting and Range and Days to Planting and Water Addition Treatment are shown.

Reproductive Trait PC1 increased with later planting dates for experimental plants from the native and invasive ranges, but showed a hump‐shaped pattern in the landrace range; these patterns are statistically significant (Figure 5a, Table 2). Reproductive Trait PC2 increased with later planting dates for experimental plants from invasive, and landrace ranges but declined in the highest water addition treatments for the native range; these patterns significantly differed among ranges (Figure 5b). Trait values of Vegetative Trait PC1 (highly loaded for leaf traits) in invasive and landrace plants peaked at a range of planting times of 10–20 d from the last frost date while values declined with later planting dates in native populations (Figure 5c). Plants had higher Vegetative Trait PC2 values when seeds were started later relative to the last average frost date (Figure 5d).

The effects of planting time on all composite traits did not differ among water addition treatments (Figure 5e–h, Table 2).

### 3.5 Amount of plasticity among source ranges (Hypothesis 3)

**FIGURE 6:**
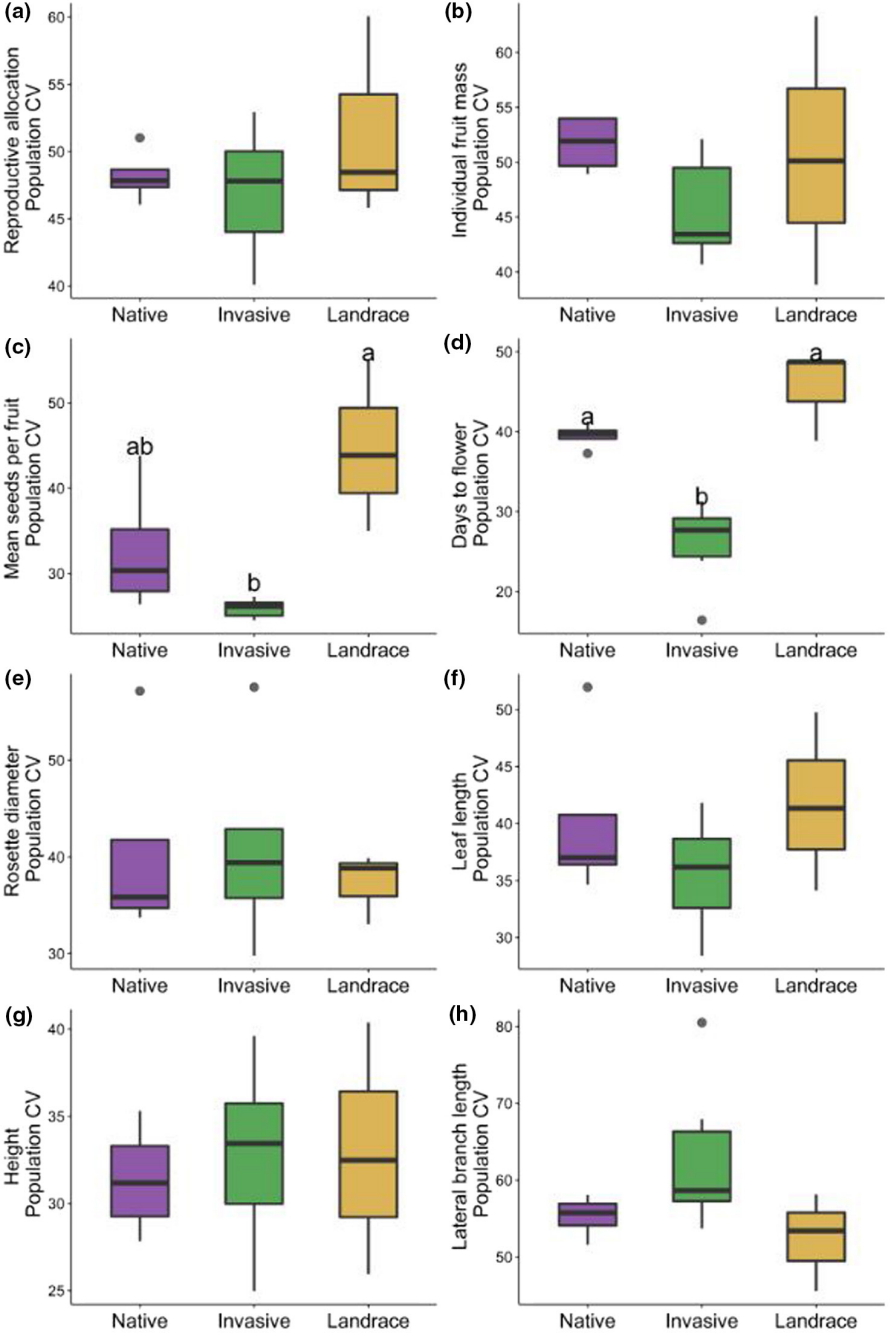
Box plots of individual trait CVs in native (*N* = 4), invasive (*N* = 7), and landrace (*N* = 3) ranges. Pairwise comparisons of traits that have significantly different means are indicated by Tukey HSD superscripts.

The mean coefficient of variation (our variable for amount of plasticity) of native, invasive, and landrace experimental populations statistically differed for mean seeds per fruit and days to flower (Figure 6 and Table 3), but not for reproductive allocation, individual fruit mass, rosette diameter, leaf length, plant height, and lateral branch length. The experimental populations sourced from invasive localities had the lowest CV for mean seeds per fruit, while the landrace group had the largest amount of plasticity for seeds per fruit (Figure 6c, Table 3). The invasive experimental plants had the lowest plasticity for flowering time, while the landrace group had the most plasticity for flowering time (Figure 6d, Table 3).

**TABLE 3:** Summary of ANOVA results that tested the differences in coefficient of variations of eight life history traits associated with reproduction, leaf morphology, and plant architecture (*n* = 14 populations per trait).

Trait CV	Source	*df*	*F*	*p*
Reproductive allocation	Range	2	0.86	.449
Individual fruit mass	Range	2	1.34	.301
Mean seeds per fruit	Range	2	10.44	**.002**
Days to flower	Range	2	23.68	**<.0001**
Rosette diameter	Range	2	0.167	.848
Leaf length	Range	2	1.242	.326
Plant height	Range	2	0.119	.889
Lateral branch length	Range	2	2.594	.119

*Note*: Bold values are the exact outputs from the statistical software.

### 3.6 Association with fitness (Hypothesis 4)

**FIGURE 7:**
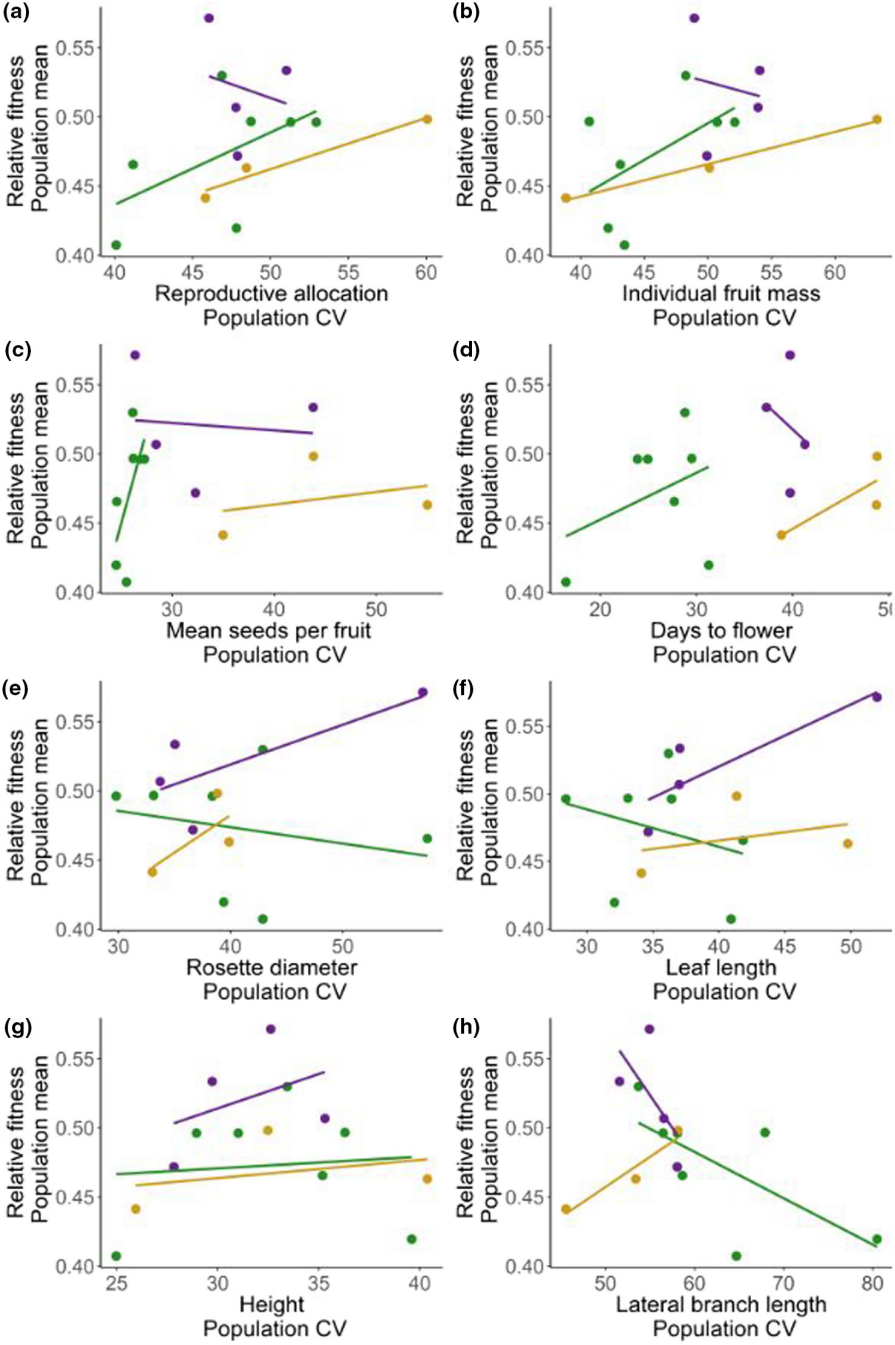
Association of individual traits versus relative fitness (Purple = Native, Green = Invasive, Yellow = Landraces).

Individual fruit mass and lateral branch length varied with relative fitness (Figure 7). Those results approached significance (Table 4).

**TABLE 4:** Summary of type III ANCOVA results that tested the relationship between population coefficient of variations of eight life history traits (*n* = 14 populations per trait) and relative fitness among native, invasive, and landrace ranges.

Source	Reproductive allocation			Individual fruit mass			Mean seeds per fruit			Days to flower			Rosette diameter			Leaf length			Height			Lateral branch length		
	*df*	*F*	*p*	*df*	*F*	*p*	*df*	*F*	*p*	*df*	*F*	*p*	*df*	*F*	*p*	*df*	*F*	*p*	*df*	*F*	*p*	*df*	*F*	*p*
Trait	1	2	.19	1	3.6	.09	1	0	.88	1	1	.34	1	0.4	.53	1	1	.36	1	0.069	.8	1	3.88	.084
Range	2	2.4	.15	2	1.3	.32	2	2.1	.19	2	1.8	.22	2	1.9	.22	2	1.8	.22	2	1.67	.24	2	2.57	.13
Trait × Range	2	0.3	.75	2	0.4	.68	2	1.5	.29	2	0.2	.82	2	1.1	.38	2	1.3	.34	2	0.10	.90	2	2.14	.17

## 4 DISCUSSION

The performance of desert‐adapted plants depends on water availability (Aparecido et al., 2020). Most of the norms of reaction that we produced for *Brassica tournefortii* varied by adding different amounts of water to the soil. Specifically, we observed different patterns of phenotypic plasticity of life history traits of experimental *B. tournefortii* plants that we artificially reproduced from native, invasive, and landrace range sources (Hypothesis 2). We observed increasing phenotypic responses for experimental plants from all ranges of sources from low to moderate water additions. We expected this result because water is a critical resource for plant development (Nicotra & Davidson, 2010). In our arid common garden, plants that received a moderate amount of water grew bigger than plants in drought conditions. However, the interpretation of the reaction norms of experimental plants from moderate to high water additions was complicated. We observed that native and invasive experimental plants showed differing patterns of phenotypic responses to moderate to high water addition compared to the landrace experimental plants. Nonetheless, even with differential water additions, some of the phenotypic variation that we observed was due to the geographic and/or genetic source of the seeds (Alfaro & Marshall, 2019). That is, the mean values of composite traits varied among experimental populations sourced from native, invasive, and landrace source ranges (Hypothesis 1). Still, we did not detect range‐by‐treatment interactions in the composite or individual traits that we examined.

In a population that experiences fluctuating resources, individual plants that can be plastic in their size and their reproductive allocation might increase their relative fitness (Doust, 1989). In resource‐poor habitats, organisms tend to be smaller to ensure survival (Venable, 1992), and this was the pattern that experimental plants exhibited in drought conditions for native‐ and invasive‐sourced *B. tournefortii*. But our experimental plants initially sourced from wild populations did not grow larger rosettes or have larger leaves and more complex leaf morphology when the soil was saturated with water. Despite this plateau in rosette and leaf trait values, the continuous addition of water resulted in a linear increase in reproductive allocation in the same experimental plants from the native and invasive ranges. A possible hypothesis to explain this limit to rosette and leaf development is that mature rosettes that reach reproductive maturity can allocate more energy and resources into bolting and reproducing in wild populations of *B. tournefortii* when rosette and leaf growth is constrained at reproduction. This could be due to transport of mobile nutrients, especially nitrogen and phosphorous, from leaves to inflorescences during the reproductive stage (Maillard et al., 2015). The behavior of invasive populations in drought and rainy years reflects the phenotypic responses of our experimental plants to the low, moderate, and high water addition treatments. Invasive U.S. populations of *B. tournefortii*, where some of our experimental lines were sourced, are known to respond positively to pulse precipitation patterns (Schwinning & Ehleringer, 2001). Specifically, *B. tournefortii* in the southwestern United States expands in patch size and range coverage when the amount and frequency of precipitation are high, but population size decreases in extended periods of drought (Li et al., 2015).

Landrace experimental plants did not change in flowering phenology, seed count, plant size, and branching (Reproductive Trait PC2 and Vegetative Trait PC2) when there was a surplus of water. This stable composite trait response to increasing watering amounts is potentially a favorable type of plasticity that can benefit seed crops like *B. tournefortii* (Husen et al., 2014). Specifically, plants with the combination of plastic traits described above can potentially optimize the conversion of water and nutrients into consistent seed yields. This pattern is absent in native and invasive experimental plants, where rosette size and leaf traits were stable with increasing water, but there was no plateau of trait values for plant height and architecture.

While planting time was not an intentional experimental variable, we include an interpretation of the phenotypic variability that may have resulted from this effect. Therefore, we evaluated the effects of planting date and considered what these effects might mean in the context of phenotypic plasticity. By planting seeds at different times, seedlings emerged at different daytime and nighttime temperatures throughout the experiment. In *Brassica* species, initial conditions during the early seedling stage can determine trait expression and plant performance in later stages (Angadi et al., 2000), as we observed in our plants. Planting date did not affect water addition treatments, but composite trait variation differed due to the interaction of planting date and source range. Reproduction and growth of our *B. tournefortii* depended on the time of seedling germination relative to the last frost date. We observed similar trends in Reproductive Trait PC1 (reproductive allocation, individual fruit mass, and number of fruits per plant) and Vegetative Trait PC2 (plant height and branch traits) in that there was a consistent linear increase in both Reproductive Trait PC1 and Vegetative Trait PC2 throughout the planting period across experimental plants derived from native and invasive populations. However, landrace plants showed nonlinear trends for Reproductive Trait PC1 and Vegetative Trait PC2. In particular, landrace seedlings planted in the middle of our planting period had optimal reproduction, as shown by a moderate hump‐shaped graph for Reproductive Trait PC1. Landrace plants that were planted later had larger sizes and more complex branching architecture (Vegetative Trait PC2).

Branch development in *Brassica* has been shown to suffer in temperatures that are 35°C or higher, but reaction to heat stress is complicated and can be improved by temperatures in earlier and later development phases (Angadi et al., 2000). A possible mechanism for growth increases in later planting dates, gradual warming during the early seedling stage may have induced resistance to heat stress in the reproductive stage, which occurs in warmer periods (Morrison & Stewart, 2002). Seedlings derived from native, invasive, and landrace populations that were started at a later date rapidly increased in Vegetative Trait PC2 values, which indicates larger plants with more complex architecture can develop when the starting conditions include warmer temperatures. In our study, this coincides with seedling stage in mid‐May and bolting in mid‐July. It is common for desert plants to have more complex branching, leaf, or spike architecture to create a boundary layer that can stabilize temperature (Gibson, 2012). We therefore hypothesize that landraces are plastic for plant size and architecture to prevent heat stress.

For Vegetative Trait PC1, which is highly loaded for rosette size and leaf morphological traits, the patterns of phenotypic variation are hump‐shaped for plants derived from native and landrace populations. These nonlinear trends indicate optimal growth when native and landrace seedlings are started in moderate temperatures in the absence of cold or heat stress. One reason is that seedlings will tend to develop poorly due to freezing and heat stress (e.g. Niu et al., 2021). Experimental plants from invasive populations have decreasing trends in Vegetative Trait PC1 values as a response to planting dates. Plants from the invasive populations in the Mojave Desert, where some of the source populations originated, are known to have seedlings that emerge early and rapidly in the growing season (Marushia et al., 2010). This early and rapid emergence increases the performance of invasive seedlings versus other non‐native invaders in the region (Marushia et al., 2012).

In addition to a broad assessment of the response of composite traits to soil water addition, we separately analyzed individual traits among experimental groups to analyze variation in the amount of plasticity (Hypothesis 3) and to test for adaptive plasticity (Hypothesis 4). For Hypothesis 3, we predicted the invasive range to have a higher mean plasticity than would native and crop ranges. For our set of experimental plants, we did not observe invasive plants to have higher amount of plasticity than native and landrace plants. Instead, landrace experimental populations showed higher mean plasticity than wild (native and invasive) experimental populations. Mean seeds per fruit and days to flower showed statistically significant differences in the amount of plasticity among ranges, even though the reaction norms of the associated composite trait (Reproductive Trait PC2, highly loaded for seed number per fruit and days to flower) show a stable pattern. We hesitate to explain further how the amount of plasticity in seed number per fruit and days to flower can be related to reaction norm patterns for the composite trait (Reproductive Trait PC2) because the reaction norm results were not statistically significant. When we analyzed the association between pooled individual trait plasticity and pooled relative fitness to test Hypothesis 4, the trends for individual fruit mass and lateral branch length approached significance. Perhaps an expanded sample from more sources from the native, invasive, and landrace populations would paint a clearer picture of the adaptive potential of plasticity (Davidson et al., 2011) via soil moisture in life history traits in this plant.

In conclusion, the reaction norms we produced show that *Brassica tournefortii* thrives in a range of soil moisture conditions in critical stages of development. We can interpret this ecological breadth as a signal for drought tolerance. Our own field observations from the invasive range confirm that some of the results we saw represent the potential behavior of these plants in the field. For instance, smaller plants were found in upland areas away from water sources, while relatively taller and more fecund plants were situated in riparian areas or irrigated land (B. Alfaro personal observation). While our samples were far removed from their original sources with few bouts of propagation, our results can still be used as a baseline to predict how this species will grow in certain environments. Crop breeders and growers can use a combination of plastic genotypes in their fields to increase yield. It could therefore be beneficial for breeders to scan and select for both stability and plasticity of ecological and agronomic traits, so growers can respond promptly to environments that may be changing too rapidly for technological and genetic development. Plant conservation and weed eradication programs should consider conducting experiments that determine plastic responses to key environmental factors. Or, field technicians can include size and reproductive trait measurements in their surveys so they can later perform a coarse‐scale assessment of phenotypic variability and potential plasticity in target populations. Such experiments and survey efforts will provide information on which native populations to select for propagation; on the other hand, an invasive weed control effort can focus on removing populations that show trait plasticity that is ecologically important.

## AUTHOR CONTRIBUTIONS

**Brian Alfaro:** Conceptualization (equal); data curation (lead); formal analysis (equal); funding acquisition (equal); investigation (equal); methodology (lead); project administration (equal); resources (equal); software (equal); supervision (supporting); validation (equal); visualization (lead); writing – original draft (equal); writing – review and editing (equal). **Diane L. Marshall:** Conceptualization (equal); formal analysis (equal); investigation (equal); methodology (equal); software (equal); supervision (equal); writing – review and editing (equal).

## FUNDING INFORMATION

Partial funding from UNM Graduate and Professional Student Association (GPSA) New Mexico Research Grant was used in this project.

## CONFLICT OF INTEREST STATEMENT

The authors declare no conflict of interest.

## Supporting information


Table S1.

## Data Availability

The data that support the findings of this study, along with relevant metadata and codes, are openly available in Dryad at https://datadryad.org/stash/share/IlKHMr3UgvodfZhxsdwiSm6C10W548iFkYJWnqG_IpA.
