# Supplementary material for: Evidence of differential phenotypic plasticity in a desert mustard
Source: Ecol Evol. 2023 Aug 31;13(9):e10479. doi: 10.1002/ece3.10479 (PMC10468984; doi:10.1002/ece3.10479)
Supplement: Supplementary file 1 — Table S1. [file ECE3-13-e10479-s001.docx]

Supplemental Table 1 – Accession information for native and landrace sources.

| **Country** | **USDA Accession No.** | **Sampling Date** | **Locality** |
| --- | --- | --- | --- |
| India, Uttar Pradesh | PI 603030 | 1998 | Uttar Pradesh, India (UTP) |
| Israel | PI 649216 | 1998 | Palmachim, Israel (PAL) |
| Morocco | PI 388764 | 1974 | Tiznit, Morroco (MOR) |
| Pakistan | PI 426416 | 1978 | Sammundri, Pakistan (SAM) |
| Pakistan | PI 426418 | 1978 | Fateh Jang, Pakistan (FAT) |
| Spain, Almeria | PI 633184 | 1996 | Nijar, Spain (NAJ) |
| Spain, Madrid | PI 296063 | 1964 | Madrid, Spain (MAD) |
